# Metabolomics and Network Pharmacology-Based Screening of Candidate Hepatoprotective Metabolites in Fermented *Dendrobium officinale* Against Acetaminophen-Induced Liver Injury

**DOI:** 10.3390/cimb48030242

**Published:** 2026-02-25

**Authors:** Haiyue Pang, Hongtan Wu, Yu Zhong, Yiheng Deng, Yadong Feng, Gueyhorng Wang, Chihli Yu

**Affiliations:** Engineering Research Center of Natural Cosmeceuticals College of Fujian Province, Department of Public Health and Medical Technology, Xiamen Medical College, Xiamen 361023, China; 201500010339@xmmc.edu.cn (H.P.); 201500010324@xmmc.edu.cn (H.W.); 15289867182@163.com (Y.Z.); yiheng_deng@163.com (Y.D.); 202300010507@xmmc.edu.cn (Y.F.)

**Keywords:** acetaminophen-induced liver injury, fermentation, *Dendrobium officinale*, *Saccharomyces cerevisiae*, antioxidant

## Abstract

*Dendrobium officinale* exhibits hepatoprotective potential against acetaminophen-induced liver injury (AILI). Fermentation has been proposed as a strategy to enhance the utilization and efficacy of herbal medicines. However, whether yeast fermentation improves the hepatoprotective effects of *D. officinale* remains unclear. This study investigated whether fermentation of *D. officinale* flower extract with *Saccharomyces cerevisiae* (1002S) enhances its protective effects against AILI, compared with a nonfermented extract (DOFE). Hepatoprotective efficacy was evaluated in male C57BL/6 mice, which received 1002S or DOFE (500 mg/kg, oral gavage) for 7 days before an acute acetaminophen challenge. Untargeted metabolomics and network pharmacology analyses were used to characterize fermentation-associated metabolic alterations and to explore potential pathways related to the observed effects. Metabolomic profiling revealed distinct metabolic differences between 1002S and DOFE. Network pharmacology analysis indicated predicted targets of fermentation-associated metabolites were associated with the phosphoinositide 3-kinase (PI3K)/ protein kinase B (Akt) and Janus kinase (JAK)/signal transducer and activator of transcription proteins (STAT) signaling pathways. In vivo, 1002S more effectively alleviated hepatocellular necrosis and significantly reduced serum alanine aminotransferase (ALT) and aspartate aminotransferase (AST) levels. Increased expression of nuclear factor erythroid 2-related factor 2 (Nrf2), superoxide dismutase 2 (SOD2), solute carrier family 7 member 11 (SLC7A11) and glutathione peroxidase 4 (GPX4) was observed in liver tissues. Molecular docking suggested hemsleyanoside may contribute to these effects. Collectively, *S. cerevisiae* fermentation enhanced the antioxidant and hepatoprotective efficacy of *D. officinale* flower extract, supporting its potential development for AILI prevention.

## 1. Introduction

Acetaminophen (APAP) is one of the most widely used antipyretic and analgesic drugs worldwide [1]. Although generally safe at therapeutic doses, APAP overdose remains the leading cause of drug-induced liver injury (DILI) and acute liver failure (ALF), particularly in Western countries [2]. The pathogenesis of APAP-induced liver injury (AILI) involves complex interactions among intracellular signaling events, including mitochondrial damage, reactive oxygen species (ROS) accumulation, and inflammatory responses [3]. At therapeutic doses, APAP is primarily detoxified via glucuronidation and sulfation in the liver. However, approximately 5–15% of APAP is oxidized by the cytochrome P450 system (especially Cytochrome P450 2E1; CYP2E1) to the active intermediate N-acetyl-p-benzoquinone imine (NAPQI) [4,5]. Excessive NAPQI formation leads to profound depletion of hepatic glutathione (GSH) [6]. Both NAPQI and ROS induce mitochondrial DNA damage and trigger the sustained activation of the c-Jun N-terminal kinase (JNK) signaling pathway, amplifying the generation of mitochondrial ROS and promoting the opening of the mitochondrial permeability transition (MPT) pore [5]. The persistent opening of the MPT pore leads to a catastrophic loss of membrane potential and mitochondrial swelling, ultimately culminating in hepatocyte necrosis [7]. The release of damage-associated molecular patterns (DAMPs), such as high mobility group box 1, activates sterile inflammatory signaling (tumor necrosis factor-alpha and interleukin-1 beta), thereby further exacerbating the initial damage [8,9].

The antioxidant defense system, primarily comprising superoxide dismutase (SOD), catalase (CAT), and glutathione peroxidase (GPx), is critical for maintaining hepatic redox homeostasis [10,11]. SOD converts superoxide radicals into hydrogen peroxide (H_2_O_2_). CAT promotes the decomposition of H_2_O_2_ into water and oxygen, while GPx reduces various peroxides to prevent lipid peroxidation. The consumption of these enzymes leads to ROS accumulation and is accompanied by an increase in malondialdehyde (MDA) levels [12]. The hepatocellular damage is reflected in elevated serum levels of ALT and AST [13]. Histopathologically, AILI is characterized by hepatocellular necrosis, inflammatory infiltration, and apoptosis, indicating a correlation between oxidative stress and liver tissue damage [14,15]. The C57BL/6 mice model effectively mimics the hepatotoxicity induced by acetaminophen in humans, providing a reliable research basis for elucidating the mechanism of liver injury and preclinical evaluation of hepatoprotective strategies [16].

Under physiological conditions, the Kelch-like ECH-associated protein 1 (KEAP1)–Nrf2 complex serves as a molecular sensor for detecting intracellular redox states [17]. APAP-induced oxidative stress has been reported to promote oxidative modification of KEAP1 cysteine residues, leading to the dissociation of Nrf2 from the KEAP1 complex and its translocation into the nucleus [18]. Nrf2 is a key transcriptional regulator of antioxidant defense, coordinating the expression of protective factors to reduce oxidative stress and maintain cellular homeostasis [19]. Recent studies have shown that ferroptosis, a regulated cell death driven by iron-dependent lipid peroxidation, plays a crucial role in the progression of AILI [20,21]. Ferroptosis is regulated by proteins, including Nrf2, cystine/glutamate transporter (SLC7A11/xCT), GPX4. The pathological progression of acute liver injury could be mediated through the activation of the Nrf2/xCT/GPX4 pathway [22,23,24]. Nrf2 also regulates the expression of SLC7A11, GSH, and GPX4 to inhibit ferroptosis [25]. GPX4, activated by glutathione, protects cells against lipid peroxidation by reducing ROS levels [26]. Increased GPX4 further prevents iron-induced cell death and maintains cellular redox homeostasis [27]. SLC7A11 indirectly affects cellular sensitivity to ferroptosis by regulating redox homeostasis and the ferroptosis inhibition pathway [28].

*Dendrobium officinale* is recognized as a homologous plant of medicine and food in China and exerts multiple biological activities, including immunomodulatory and antioxidant effects [29]. It contains a wide range of bioactive compounds, including polysaccharides, flavonoids, phenolics, and organic acids [30]. Its polysaccharides can activate leukocytes [31], enhance immune function [32], exhibit anti-tumor potential [33] and provide protective effects against AILI [34]. Otherwise, fermentation has emerged as a biotechnological strategy to enhance the therapeutic efficacy and bioavailability of traditional herbs [35]. In particular, yeast fermentation, such as that by *S*. *cerevisiae*, can induce specific biochemical transformations, including glycoside hydrolysis, structural modification of phenolic compounds, and redox-related reactions, thereby altering metabolite profiles and enhancing antioxidant and anti-inflammatory activities [36,37,38]. The fermentation products may modulate key pathways involved in oxidative stress and ferroptosis, offering a potential therapeutic avenue for mitigating AILI [39]. However, the complexity and abundance of compounds in fermentation products make it challenging to determine which specific constituents exert protective effects. 

Previous studies have reported that the fermentation process can significantly enhance the antioxidant activity and active ingredients of *D. officinale* [40]. Given that oxidative stress and redox imbalance play central roles in AILI, these fermentation-induced metabolic changes may be related to hepatoprotection. However, due to the chemical complexity of fermentation products, the specific metabolites and pathways responsible for potential protective effects remain unclear.

In the field of traditional Chinese medicine, network pharmacology is a widely used computational approach to predict potential targets and pathways of bioactive compounds based on chemical structure and curated databases [41]. Integrating network pharmacology with metabolomics provides a systems-level framework to associate candidate bioactive compounds with predicted signaling pathways [42]. Molecular docking is a computational technique used to predict the binding modes and affinities of ligands to receptor proteins, including small molecules, peptides, antibodies, and nucleic acid inhibitors [43,44]. While the strategy facilitates hypothesis generation regarding multi-target interactions, it does not replace experimental validation.

Therefore, in this study, a yeast-fermented *D. officinale* flower extract was prepared, and its hepatoprotective effects were first evaluated in AILI models. Integrated metabolomics and network pharmacology analyses were subsequently applied to characterize fermentation-associated metabolic alterations and to investigate potential bioactive compounds and signaling proteins that may contribute to the observed effects. The overall aim of this study was to elucidate the hepatoprotective effects and associated mechanisms of fermented *D. officinale*, particularly in relation to redox homeostasis and ferroptosis-associated pathways, thereby providing a scientific basis for its potential development as a functional food or therapeutic candidate for the prevention and treatment of AILI.

## 2. Materials and Methods

### 2.1. Materials

*S. cerevisiae* (CICC 1002) was purchased from the China Center of Industrial Culture Collection (Shanghai, China). Kits of 2,2′-azino-bis(3-ethylbenzothiazoline-6-sulfonic acid) (ABTS), 3-(4,5-dimethylthiazol-2yl)-2,5-diphenyltetrazolium bromide (MTT) and ferric reducing antioxidant power (FRAP) were from Beyotime Biotech Inc. (Shanghai, China). 2,2-diphenyl-1- picrylhydrazyl (DPPH) and paracetamol were from Sigma-Aldrich (St. Louis, MO, USA). TRIzolTM Reagent was from Thermo Fisher Scientific (Waltham, MA, USA). Dulbecco’s Modified Eagle’s Medium (DMEM), fetal bovine serum (FBS), penicillin, and streptomycin were from Invitrogen (Carlsbad, CA, USA). Kits of ALT, AST, SOD, CAT, GSH, GSH-Px, and MDA were from Nanjing Jiancheng Institute of Biotechnology (Nanjing, China). Antibodies to Nrf2, SOD2, SLC7A11 and GPX4 and β-actin were from ABclonal Biotechnology (Wuhan, China), and HRP-conjugated secondary antibodies to rabbit IgG (7074) were from Jackson ImmunoResearch Laboratories (West Grove, PA, USA). All other chemicals and reagents were analytical grade and commercially available.

### 2.2. Preparation of Water and Fermented Extracts from D. officinale Flowers

*D. officinale* flowers were purchased from Yueqing Xianfeng Lanyun Dendrobium Co., Ltd., in Yueqing, Zhejiang, identified by professor Hongjuan Bao (Xiamen Medical College, Xiamen, China). A voucher specimen was deposited in the Department of Pharmacy, Xiamen Medical College.

Fresh *D. officinale* flowers were pulverized and sterilized at 121 °C for 30 min. The resulting paste was split into two parts: one part was extracted using distilled water (1:5, w/v) to prepare DOFE, and the other part was fermented with 5% *S. cerevisiae* at 37 ℃ for 96 h to obtain 1002S. The mixtures were subjected to ultrasonic extraction (585 W, 30 min, twice), filtered, concentrated using a rotary vacuum evaporator (Evela, Tokyo, Japan) at 55 °C, freeze-dried (Evela, Tokyo, Japan), and resuspended in water for use.

### 2.3. Measurement of Antioxidant Activities In Vitro

DPPH radical scavenging activity was determined by mixing 20 μL of sample solution with 180 μL of 100 μM DPPH in methanol. The mixture was incubated in the dark at 37 °C for 30 min, and absorbance was measured at 517 nm. ABTS+ radical scavenging activity was measured using a commercial assay kit. Briefly, 20 μL of the sample was mixed with 180 μL of ABTS working solution, incubated in the dark for 10 min, and the absorbance was recorded at 734 nm.

The radical scavenging rates were calculated using the following formula:

$$Radical\;scavenging\;rate\;(\%)=\frac{\left(A0-A1\right)}{A0}\times 100\%$$

A0: Absorbance value of the blank solution;

A1: Absorbance value of the sample solution.

Dose–response curves were constructed for each sample, and IC_50_ values were calculated by nonlinear regression analysis using GraphPad Prism 10 (GraphPad Software, San Diego, CA, USA). Ascorbic acid (for DPPH assay) and Trolox (for ABTS+ assay) were used as reference antioxidants.

Ferric reducing antioxidant power (FRAP) was determined using a commercial kit. Briefly, 20 μL of the sample was mixed with 180 μL of FRAP reagent and incubated in the dark for 30 min. Absorbance was recorded at 595 nm, and antioxidant capacity was calculated from a FeSO4 standard curve (500–2000 μM), expressed in mM FeSO_4_.

### 2.4. Cell Culture and Treatment

HepG2 cells, a hepatoblastoma cell line, are widely used as human hepatocyte-derived models for studying oxidative stress-related cellular responses [22]. HepG2 cells were cultured in DMEM high-glucose medium supplemented with 10% FBS, 2 mM L-glutamine, 100 units/mL penicillin, and 100 μg/mL streptomycin at 37 ℃ in a humidified incubator with a 5% CO_2_-enriched atmosphere. 

Cell viability was measured by the MTT assay. Cells (2 × 10^4^ cells/well) in 96-well plates were pretreated with DOFE or 1002S samples (0.2−0.05 mg/mL) for 24 h. After treatment, 10 μL of MTT solution (5 mg/mL) was added and incubated for 4 h. The medium was removed, the formazan crystals were dissolved in 150 μL of DMSO and the absorbance was measured at 490 nm.

To assess the antioxidant effects under APAP-induced oxidative stress, cells (2 × 10^4^ cells/well) were pretreated with samples for 2 h, then exposed to 300 μM APAP for 20 h without medium change. The control group received medium only, and the model group received APAP only. Cell viability was measured by CCK8 and expressed as a percentage of the control.

### 2.5. Animals and Experimental Design

A total of 36 male C57BL/6 mice, 6–8 weeks old, 20 ± 2 g weight, were purchased from the Laboratory Animal Center, Xiamen University (Xiamen, China). The mice were maintained with free access to water and food in the animal room with a constant temperature of 22 ± 2 °C and a 12 h light–dark cycle. Before the experiment, all mice were given a 7-day acclimatization period to stabilize their physiological state. No specific strategies were applied to minimize potential confounders such as cage location or measurement order. Each experimental group consisted of a total of nine mice (*n* = 9). These animals were derived from three completely independent experiments conducted on separate occasions using different batches of mice, with three mice per group in each experiment. Each mouse was treated as an independent biological replicate, and all statistical analyses were performed at the individual animal level rather than on averaged subgroup values. 

The mice were randomly divided into four groups: control, paracetamol-intoxicated (APAP), DOFE, and 1002S groups. Oral administration of acetaminophen was used to induce acute liver injury. A relatively high dose of APAP was selected to establish a robust and reproducible model of acute hepatotoxicity within a defined experimental time window. Although this dose is higher than that used in some studies, oral APAP administration at high doses has been widely reported to reliably induce severe liver injury in mice, particularly when rapid and consistent hepatocellular necrosis is required for mechanistic and intervention studies. The DOFE and 1002S groups were administered orally at a dose of 500 mg/kg body weight once daily for 7 days, while the control and APAP groups received saline. Two hours after the final dose, the APAP, DOFE, and 1002S groups received 500 mg/kg paracetamol orally; the control group received saline. Six hours later, the mice were euthanized by CO_2_ inhalation, and the livers were collected. Serum was separated by centrifugation (10,000× *g*, 4 °C) and stored at −80 °C. Liver tissues were rinsed with ice-cold PBS; one portion was fixed in 4% paraformaldehyde for histology, and the other was flash-frozen in liquid nitrogen and stored at −80 °C for further analysis. No animals/data were excluded from the analysis. As this study was exploratory, a primary outcome measure was not defined. The sample size was based on previous studies and practical considerations [33,45]. Formal a priori power calculations were not performed. Sample sizes were determined based on previously published studies using acetaminophen-induced liver injury models while adhering to ethical considerations and the principles of the 3Rs (Replacement, Reduction, and Refinement) for animal research. The mice were monitored every 2 h for clinical signs of distress. Humane endpoints were predefined as signs of severe respiratory distress.

### 2.6. Histopathological Examination of Liver Tissue

Liver tissues were washed with 1 × PBS, dehydrated, and embedded in paraffin. Sections (5 μm) were prepared and stained with hematoxylin and eosin (H&E). After staining, the slides were dehydrated through a graded series of ethanol, cleared in xylene, and mounted with neutral resin. Images were captured using a light microscope (Nikon, Tokyo, Japan) equipped with a digital camera. The histopathological changes, including hepatocyte necrosis and inflammatory infiltration, were evaluated by an experienced pathologist blinded to the group assignments. To further quantify hepatic inflammatory infiltration, histological images were analyzed using ImageJ software (National Institutes of Health, Bethesda, MA, USA). Color deconvolution was applied to separate hematoxylin and eosin signals, and the area of inflammatory cell infiltration was quantified in representative fields. The results were expressed as a semi-quantitative measure of hepatic inflammation.

### 2.7. Measurement of Biochemical Parameters in Livers

Liver tissues were homogenized in ice-cold saline (1:9, w/v) and centrifuged at 3000 rpm for 10 min at 4 °C. The resulting supernatant was collected and used to measure AST, ALT and SOD activities by commercial kits. Total proteins were determined using the BCA assay.

ALT catalyzes the transfer of an amino group from L-alanine to α-ketoglutarate, producing pyruvate. The pyruvate reacts with the kit’s chromogenic reagent to generate a colored product proportional to enzyme activity. Samples were incubated with ALT reagent at 37 °C, and absorbance at 505 nm was used to calculate activity (U/L) from kit standards. AST catalyzes the transfer of an amino group from L-aspartate to α-ketoglutarate, forming oxaloacetate. Oxaloacetate reacts with the chromogenic reagent in the kit, producing a color whose intensity is proportional to AST activity. Samples were incubated with AST reagent at 37 °C, and absorbance at 505 nm was used to calculate activity (U/L) from kit standards. SOD activity was determined by its ability to inhibit the formation of WST-1 formazan from superoxide radicals, with absorbance at 450 nm compared to an uninhibited control. CAT activity was determined by measuring the decomposition rate of H_2_O_2_ at 240 nm. Reduced GSH levels were quantified using a colorimetric assay based on the reaction between GSH and 5,5′-dithiobis-(2-nitrobenzoic acid) (DTNB). The absorbance was measured at 412 nm using a microplate reader. GSH-Px activity was assessed by monitoring the rate of GSH oxidation during the reduction of peroxides. MDA levels, as an index of lipid peroxidation, were measured using the thiobarbituric acid reactive substance (TBARS) method. All assays were performed using commercial detection kits following the manufacturers’ protocols. All assays were performed in triplicate. The results were normalized to protein concentration, which was determined using the bicinchoninic acid (BCA) method. For biochemical assays and data analysis, samples were coded so that investigators were unaware of group allocation during measurement and analysis.

### 2.8. Quantitative Real-Time PCR Analysis

Total RNA was extracted from mouse liver tissues using TRIzol reagent (Invitrogen, Life Technologies, CA, USA). RNA purity and concentration were determined using a NanoDrop 2000 spectrophotometer (Thermo Scientific, MA, USA). Complementary DNA was synthesized by SweScript All-in-One RT SuperMix for qPCR (Servicebio, Wuhan, China). Quantitative real-time PCR (qRT-PCR) was performed with 2 × Universal Blue SYBR Green qPCR Master Mix (Servicebio, Wuhan, China) on a CFX Manager system (Bio-Rad Laboratories, Inc., Hercules, CA, USA). GAPDH served as the internal reference gene. Relative gene expression levels were calculated using the 2^−ΔΔCt^ method. The primer sequences used in this study are listed in Table S1.

### 2.9. Western Blot Analysis in Cells and Liver

Western blot analysis was conducted as described previously. Protein samples (10 μg) were separated by sodium dodecyl sulfate–polyacrylamide gel electrophoresis and transferred onto polyvinylidene difluoride membranes (PVDF). PVDF membranes were blocked in 5% BSA prepared in Tris-buffered saline containing 0.1% Tween-20 and then incubated overnight at 4 °C with primary antibodies diluted 1:3000. After three washes with TBST, the membranes were incubated with HRP-conjugated secondary antibodies (1:5000) for 1 h at room temperature. Protein bands were visualized using NcmECL Ultra chemiluminescent reagent (Suzhou, China) and detected with the ChemiDoc™ XRS+ imaging system (Bio-Rad, CA, USA). Band intensities were quantified using ImageJ software (version 1.53k; NIH, Bethesda, MD, USA), normalized to the internal loading control, and expressed relative to the control group, which was set to 100%. * indicates *p* < 0.05 compared with the control group.

### 2.10. Metabolomics Analysis

A 1 mL sample was loaded onto an SPE column, and 3 mL of methanol eluate was collected and dried under nitrogen. The residue was reconstituted in 400 μL of pre-cooled methanol–water (4:1), vortexed for 1 min, ultrasonicated in an ice-water bath for 10 min, and precipitated at −40 °C for 2 h. After centrifugation (12,000 rpm, 4 ℃, 10 min), 150 μL of the supernatant was stored at −80 °C for LC–MS, and another 150 μL was freeze-dried for GC–MS. For derivatization, the dried extract was incubated with 80 μL methoxyamine–pyridine (15 mg/mL, 37 °C, 60 min), followed by 50 μL BSTFA, 20 μL n-hexane, and 10 μL internal standard mix (C8, C9, C10, C12, C14, C16, C18, C20, C22, C24, all prepared in chloroform), then reacted at 70 °C for 60 min and left at room temperature for 30 min before GC–MS analysis. Quality control (QC) samples were prepared by pooling equal aliquots from all extracts.

All extractions were pre-cooled at −20 °C before use. Metabolomics profiling was performed using a mass spectrometry platform consisting of a Waters ACQUITY UPLC I-Class Plus system coupled with a Thermo QE HF high-resolution mass spectrometer and an Agilent 7890B-5977B GC-MS system.

For LC–MS, samples were separated on an ACQUITY UPLC HSS T3 column (100 × 2.1 mm, 1.8 µm) using water (0.1% formic acid) and acetonitrile with the following gradient: 0–2 min, 5% B; 2–4 min, 5–30% B; 4–8 min, 30–50% B; 8–10 min, 50–80% B; 10–14 min, 80–100% B; 14–15 min, 100% B; 15–15.1 min, 100–5% B; and 15.1–16 min, 5% B. The flow rate was 0.35 mL/min, the column temperature was 45 °C, and the injection volume was 2 µL. MS data were acquired in full-scan mode (70–1,050 m/z).

For GC–MS, samples were separated on a DB-5MS capillary column (30 m × 0.25 mm × 0.25 μm, Agilent J&W Scientific, Folsom, CA, USA). Helium (1.0 mL/min) served as the carrier gas. The injection was 1 µL (splitless) at 260 ℃. The program was: 60 °C for 0.5 min; ramp to 210 °C at 8 ℃/min; to 270 °C at 15 ℃/min; and to 305 °C at 20 ℃/min, hold 5 min. The electron bombardment ionization (EI) source was used at 70 eV in SCAN mode (50−500 m/z), with a solvent delay of 6.2 min. The capillary and auxiliary heater temperatures were 320 °C and 350 °C. The MS full scan range was 70–1050 m/z. 

Representative raw chromatograms for all biological replicates (*n* = 6 per group) acquired by LC-MS (positive and negative modes) and GC-MS are provided in Figures S1–S3, respectively.

### 2.11. Network Pharmacology Analysis

The molecular structures of the 25 active ingredients were identified using PubChem (https://pubchem.ncbi.nlm.nih.gov/, accessed on 1 June 2025). To explore and aggregate targets associated with “oxidative stress, liver injury, acute immune liver injury and ferroptosis”, the target proteins associated with active constituents were screened with SwissTargetPrediction (http://www.swisstargetprediction.ch/, accessed on 31 July 2025) and PharmMapper (http://www.lilab-ecust.cn/, accessed on 31 July 2025), with species set to Homo sapiens. Using antioxidant-related keywords, the targets for Homo sapiens were collected from GeneCards (https://www.genecards.org/, accessed on 31 July 2025). Target name standardization and gene symbol conversion were performed using UniProt (http://www.uniprot.org/, accessed on 31 July 2025). 

Venny 2.1 (https://bioinfogp.cnb.csic.es/tools/venny/, accessed on 31 July 2025) was used to identify intersectional targets between components and diseases as potential antioxidant drug targets. The targets were imported into STRING (https://version-11-0.string-db.org/, accessed on 31 July 2025) (Homo sapiens), and the resulting protein–protein interaction (PPI) network was analyzed in Cytoscape 3.8.0. Key genes were identified using the CytoHubba plugin, with the top 10 ranked by maximal clique centrality (MCC) scores.

The top 10 key targets were analyzed for Gene Ontology (GO) and Kyoto Encyclopedia of Genes and Genomes (KEGG) pathway enrichments using the Database for DAVID 6.8 tool (https://davidbioinformatics.nih.gov/, accessed on 31 July 2025). Results with *p* < 0.05 were considered significant. The top 20 KEGG pathways and the top 10 GO enrichments from biological process (BP), cellular component (CC), and molecular function (MF) were selected and imported into bioinformatics (https://www.bioinformatics.com.cn/, accessed on 31 July 2025). The GO enrichment analysis was selected to construct a bubble chart. A bar chart was drawn based on the KEGG pathway enrichments.

The 3D structures of ligands were obtained from PubChem, and high-resolution protein structures were obtained from RCSB PDB (http://www.rcsb.org/, accessed on 30 November 2025). Proteins were preprocessed in PyMOL (http://www.pymol.org, accessed on 30 November 2025) by removing water, phosphate groups, and other non-essential components, and docking grids were defined around active sites. PDB files were converted to PDBQT using AutoDockTools 1.5.7 (The Scripps Research Institute, La Jolla, CA, USA). Docking was performed with AutoDock Vina 1.1.2 (The Scripps Research Institute, La Jolla, CA, USA), and the highest-affinity conformation was selected [46]. Ligand–residue interactions were visualized in 2D and 3D using PyMOL and Discovery Studio 2019. 

LC-MS and GC-MS data were processed using Progenesis QI 3.0 and MS-DIAL 4.24 to generate initial data matrices. Metabolites were identified based on retention time, exact mass, fragmentation patterns, and isotopic distribution using the HMDB, Lipidmaps 2.3, METLIN, LuMet-Plant 3.0, and LuMet-GC 5.0 databases. Principal component analysis (PCA) and orthogonal partial least squares discriminant analysis (OPLS-DA) were performed via MetaboAnalyst 6.0 to visualize metabolic differences, with model quality assessed by seven-fold cross-validation and 200-response permutation testing. Data preprocessing included peak picking, missing value imputation (50% missing peaks removed; remaining zeros filled with half the minimum value), log2 transformation, and score filtering (LC-MS/MS ≥ 36, GC-MS ≥ 70). GC-MS data were normalized using internal standards and total peak area; LC-MS/MS data were scored and assigned confidence levels (1–4), with Level 1 metabolites used for functional validation. Filtered LC-MS positive/negative modes and GC-MS results were integrated into a unified matrix for pathway analysis and biomarker screening.

### 2.12. Statistical Analysis

For animal experiments, a total of 36 mice were used (4 groups, *n* = 9 mice per group). The animal study was conducted in three independent experimental batches performed at different times, with three mice per group in each batch, to ensure reproducibility. Each mouse was treated as an independent biological replicate for statistical analysis. Data from all mice within the same experimental group (total *n* = 9 per group) were pooled for statistical comparisons. The experimental batch was not used as a statistical unit but served to confirm the consistency of the observed effects across independent experiments. 

All quantitative data are presented as mean ± standard deviation (SD). 

Prior to statistical analysis, data distributions and residuals were visually inspected and found to be compatible with parametric testing.

For comparisons among multiple groups, one-way analysis of variance (ANOVA) followed by Tukey’s post hoc test was applied. 

For comparisons between two groups, Student’s t-test was used where appropriate. 

A *p*-value < 0.05 was considered statistically significant.

Statistical analyses were performed using GraphPad Prism 10 (San Diego, CA, USA).

## 3. Results

### 3.1. Determination of Antioxidant Activities In Vitro

Previous studies indicated that fermented products may exhibit stronger antioxidant activity than their unfermented extract [35]. To preliminarily compare the antioxidant-related properties of DOFE and 1002S, in vitro chemical antioxidant assays were used.

**Figure 1:**
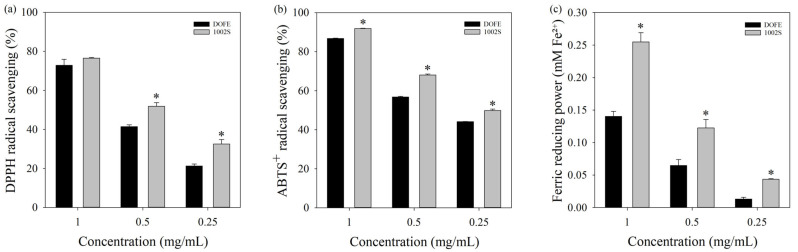
Free radical scavenging activity and ferric reducing antioxidant power. (**a**) DPPH radical scavenging activity; (**b**) ABTS+ scavenging activity; (**c**) FRAP values. Data are expressed as mean ± SD of three independent experiments performed in triplicate. Statistical significance was determined by Student’s *t*-test. * *p* < 0.05 (DOFE vs. 1002S).

Within the concentration range of 0.25–1 mg/mL, DOFE and 1002S showed dose-dependent increases in DPPH and ABTS^+^ radical scavenging activity (Figure 1a,b). The highest activities were observed at 1 mg/mL, and 1002S exhibited stronger DPPH and ABTS^+^ scavenging than DOFE. Quantitative analysis further revealed that the IC_50_ value of 1002S for DPPH radical scavenging was 0.476 ± 0.030 mg/mL, compared with 0.636 ± 0.020 mg/mL for DOFE. Ascorbic acid, used as a positive control, exhibited a markedly lower IC_50_ value of 0.038 ± 0.005 mg/mL. 

For the ABTS+ assay, the IC_50_ values were 0.253 ± 0.010 mg/mL for 1002S and 0.366 ± 0.004 mg/mL for DOFE, whereas Trolox showed a substantially lower IC_50_ of 0.032 ± 0.005 mg/mL.

The FRAP (ferric reducing antioxidant power) assay revealed that 1002S exhibited greater ferric reducing capacity than DOFE (Figure 1c). These data suggested that fermentation was associated with enhanced relative chemical antioxidant capacity under the tested conditions. 

### 3.2. Effects of DOFE and 1002S on Cell Viability

**Figure 2:**
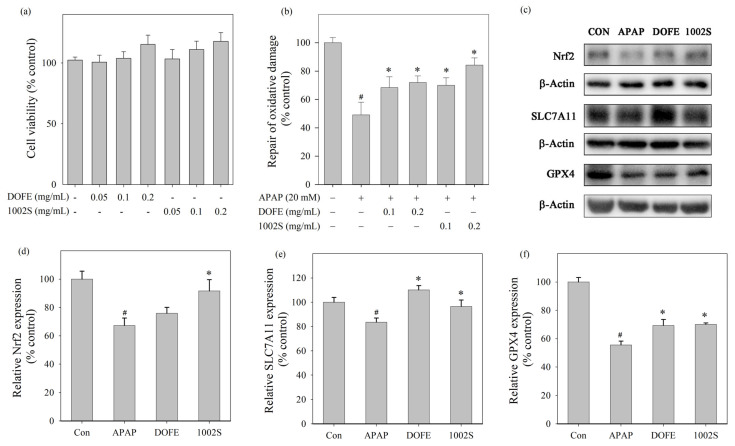
Effects of DOFE and 1002S on APAP-induced cellular injury in HepG2 cells. (**a**) Cell viability following treatment with DOFE or 1002S. (**b**) Protective effects of DOFE and 1002S against APAP-induced oxidative cytotoxicity, as assessed by cell viability. (**c**) Western blot bands of Nrf2, GPX4, and SLC7A11. (**d**–**f**) Densitometric quantification of Nrf2, GPX4, and SLC7A11 protein expression levels normalized to β-actin. Data are expressed as mean ± SD (*n* = 3). Statistical significance was determined by one-way ANOVA followed by Tukey’s post hoc test. * *p* < 0.05 vs. the APAP group. # *p* < 0.05 vs. the control group.

HepG2 cells were used in subsequent experiments to examine whether DOFE and 1002S could modulate redox- and ferroptosis-associated protein expression. The MTT assay was utilized to determine appropriate concentrations of DOFE and 1002S in HepG2 cell viability. Figure 2a illustrates that DOFE and 1002S exhibited no cytotoxic effects in HepG2 cells under 0.2 mg/mL.

DOFE and 1002S exhibited protective effects against APAP-induced oxidative damage in HepG2 cells within the concentration range of 0.1−0.2 mg/mL (Figure 2b). At 0.2 mg/mL, 1002S showed higher cell viability than DOFE, indicating stronger protective effects against APAP-induced oxidative damage. Western blot analysis (Figure 2c) showed the protein expression levels of Nrf2, GPX4 and SLC7A11. As shown in Figure 2d–f, treatment with 1002S treatments upregulated these proteins, comparable with the APAP group. The upregulation of Nrf2, GPX4, and SLC7A11 suggests that 1002S may exert its protective effects, at least in part, through modulation of redox-related and ferroptosis-associated protein expression, rather than direct ferroptosis inhibition.

### 3.3. DOFE and 1002S Alleviated AILI

**Figure 3:**
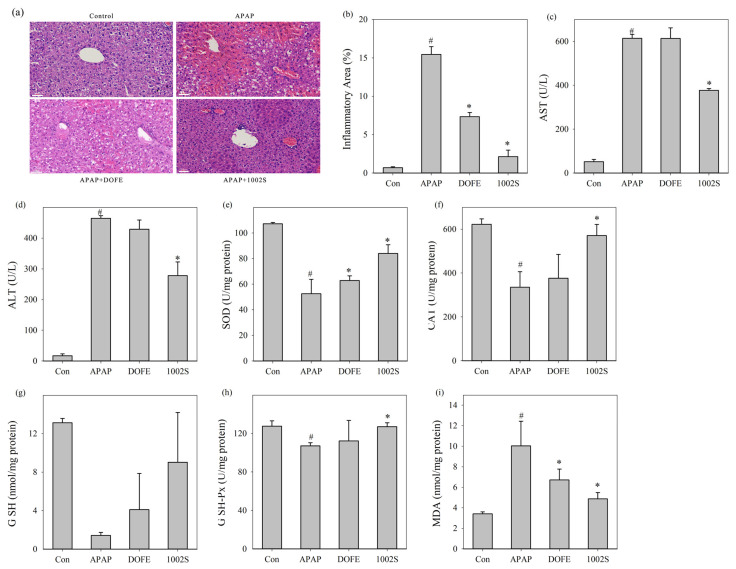
Effects of DOFE and 1002S on liver function markers and related gene expression in APAP-induced liver-injured mice. (**a**) H&E-stained liver tissue sections (10× magnification); (**b**) quantitative analysis of hepatic inflammation; (**c**) serum AST levels; (**d**) serum ALT levels; (**e**) SOD activity; (**f**) CAT activity; (**g**) GSH levels; (**h**) GSH-Px.activity; and (**i**) MDA levels. Data are expressed as mean ± SD (*n* = 9 mice per group). * *p* < 0.05 vs. the APAP group. # *p* < 0.05 vs. the control group.

To further evaluate hepatic histopathological changes, H&E staining was performed and subjected to semi-quantitative analysis (Figure 3a,b). Histological images were analyzed using ImageJ software with the Color Deconvolution plugin to quantify inflammatory infiltration areas. It should be noted that the acetaminophen dose used in this study was relatively high compared with some reports. This dose was selected to ensure consistent and pronounced liver injury within a short experimental period, rather than to model typical clinical overdose exposure.

Similar to He’s report [47], the APAP group exhibited severe liver injury, characterized by extensive necrosis and inflammatory cell infiltration in the centrilobular region. In contrast, the DOFE and 1002S groups showed attenuated liver damage, with only mild hepatocellular necrosis observed. Quantitative analysis further confirmed that both DOFE and 1002S significantly alleviated APAP-induced hepatic inflammation. The 1002S group showed more protective effects than DOFE.

Liver injury leads to ALT and AST release into the bloodstream, which are widely used clinical indicators of hepatocellular damage [48]. In Figure 3c,d, serum ALT and AST levels were significantly increased in the APAP group, indicating substantial hepatocyte injury, consistent with Tao’s result [34]. DOFE treatment can moderately reduce ALT and AST levels, whereas 1002S can significantly reduce both levels. The decreased levels of ALT and AST indicated that 1002S effectively attenuated the hepatocellular injury triggered by APAP overdose.

SOD, CAT, GSH and GSH-Px played critical roles in maintaining cellular redox equilibrium by scavenging ROS and limiting oxidative damage [10,12]. As shown in Figure 3e–i, the APAP group exhibited decreased activities of SOD, CAT, GSH, and GSH-Px, while MDA levels increased. In contrast, 1002S and DOFE reversed these changes induced by APAP, indicating enhanced antioxidant defense capabilities. Collectively, these findings suggested that 1002S attenuated AILI by reducing hepatocyte necrosis, lowering serum transaminase levels, and improving endogenous antioxidant status. These improvements collectively indicated that the liver and the endogenous antioxidant defense were restored in the AILI mice.

### 3.4. Expression of Nrf2-Associated Antioxidant and Ferroptosis-Related Proteins in Liver Tissue

**Figure 4:**
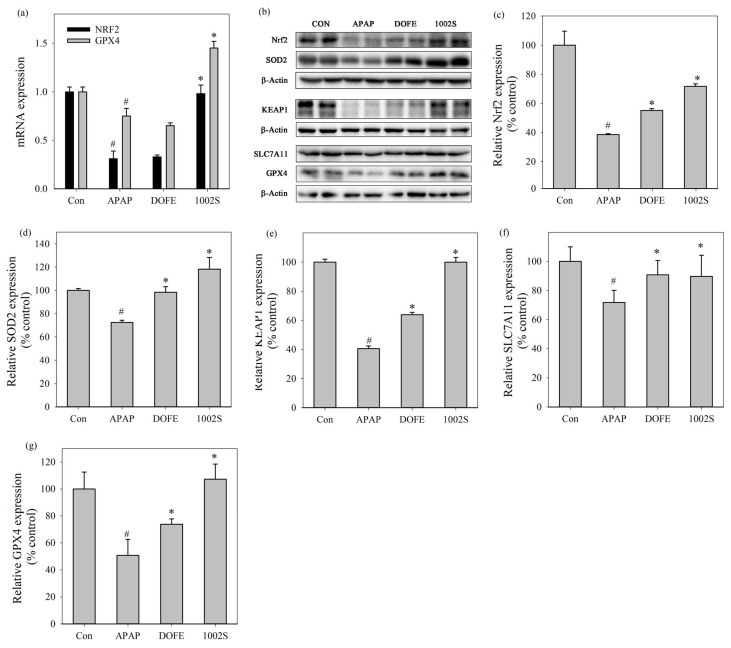
Effects of DOFE and 1002S on APAP-induced oxidative stress in liver tissues. (**a**) Hepatic Nrf2 and GPX4 mRNA expression. (**b**) Western blot bands of Nrf2, SOD2, KEAP1, SLC7A11, and GPX4. (**c**–**g**) Densitometric quantification of Nrf2, GPX4, KEAP1, SLC7A11, and GPX4 protein expression levels normalized to β-actin. Data are expressed as mean ± SD (*n* = 9 mice per group). Statistical significance was determined by one-way ANOVA followed by Tukey’s post hoc test. * *p* < 0.05 vs. the APAP group. # *p* < 0.05 vs. the control group.

To further elucidate the molecular mechanisms underlying the hepatoprotective effects, the mRNA expression levels of Nrf2 and GPX4 in liver tissues were quantified by qRT-PCR (Figure 4a). Similar to the previous finding [49], APAP administration significantly decreased the mRNA expression of Nrf2 and GPX4, indicating suppression of antioxidant defense and dysregulation of ferroptosis-related regulatory processes. In contrast, 1002S significantly restored the transcriptional levels of Nrf2 and GPX4 compared with the APAP group. 

Western blot analysis (Figure 4b–f) showed that the protein expression levels of Nrf2, SOD2, KEAP1, SLC7A11 and GPX4 were significantly reduced in the APAP group compared with the control group. In contrast, the DOFE and 1002S treatments enhanced the expressions of these proteins compared with the APAP group, with the 1002S group showing better effects than the DOFE group. 

APAP overdose induces sustained oxidative stress and mitochondrial dysfunction, which represent central pathogenic events in AILI [7]. Previous studies demonstrated that sustained oxidative injury caused by APAP exposure can simultaneously reduce Nrf2 protein expression and promote ferroptosis in liver tissues [22,50]. *D. officinale* exerted hepatoprotective effects in association with modulation of Nrf2–KEAP1–related antioxidant responses [33,51]. Moreover, the Nrf2/SLC7A11/GPX4 axis was considered an important regulatory network involved in reinforcing cellular antioxidant capacity and limiting ferroptosis under oxidative stress conditions [24,25,52]. AILI markedly suppressed SOD2 expression, leading to excessive mitochondrial ROS accumulation and aggravated oxidative damage, whereas restoration of SOD2 contributed to improved antioxidant defense and hepatoprotection [53]. GPX4 serves as a key ferroptosis-suppressing enzyme by preventing lipid peroxidation-mediated cell death, thereby playing an essential role in protecting hepatocytes under oxidative stress conditions [54]. In line with these observations, the present findings suggested that the hepatoprotective effects of 1002S are associated with restoration of Nrf2-related antioxidant responses and enhancement of ferroptosis-associated defense mechanisms, rather than definitive evidence of Nrf2 pathway activation. 

APAP administration resulted in a marked reduction in KEAP1 protein abundance, which is likely attributable to extensive hepatocellular injury and global impairment of protein stability under severe oxidative stress, rather than canonical regulation of the Keap1–Nrf2 axis. Treatment with 1002S effectively restored KEAP1 protein levels, suggesting an overall recovery of cellular redox homeostasis and protein integrity. However, as nuclear translocation of Nrf2 was not directly assessed, the present data reflect changes in the expression of Nrf2-associated components rather than direct demonstration of canonical Nrf2 pathway activation.

### 3.5. Untargeted Metabolomic Analysis

**Figure 5:**
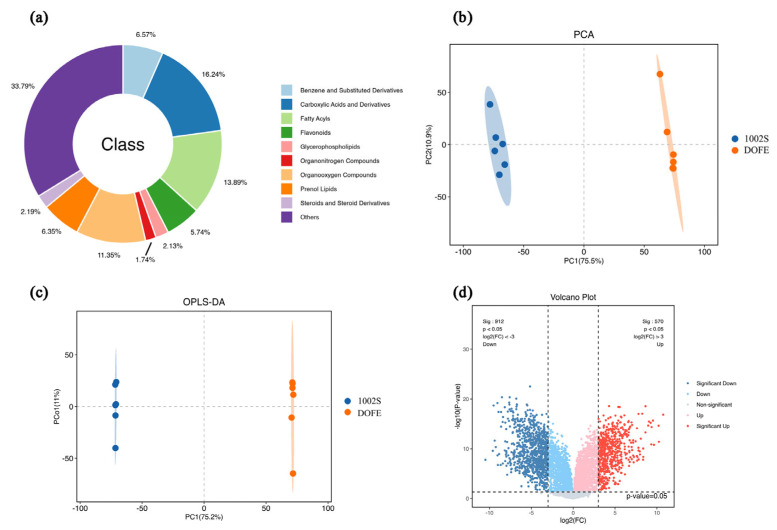
Metabolomic analysis of 1002S and DOFE. (**a**) Classification statistics of identified metabolites; (**b**) principal component analysis (PCA) of metabolite profiles between 1002S and DOFE groups; (**c**) OPLS-DA plot comparing the 1002S and DOFE groups; (**d**) volcano plot illustrating differential metabolites between groups based on fold change (FC > 3 or <1/3) and *p* < 0.05. Red and blue dots represent upregulated and downregulated metabolites, respectively.

To elucidate fermentation-associated metabolic alterations underlying the enhanced antioxidant activity of DOFE, a multiplatform untargeted metabolomics strategy integrating LC-MS/MS and GC-MS was employed to comprehensively profile the metabolic features in 1002S. Following peak detection, alignment, normalization, and quality control filtering, a total of 7227 metabolic features (m/z–RT pairs) were detected. These features were classified into ten major categories based on database annotations: benzenes and substituted derivatives, carboxylic acids and derivatives, fatty acyls, flavonoids, glycerophospholipids, organic nitrogen compounds, organic oxygen compounds, prenol lipids, steroids and steroid derivatives, and others (Figure 5a).

All detected metabolites were analyzed using multivariate statistical methods to characterize the metabolic alterations induced by fermentation. Principal component analysis (PCA) revealed a clear differentiation between the fermented and unfermented samples (Figure 5b). PC1 and PC2 explained 75.5% and 10.9% of the total variance, while PC3 explained 2.79% of the variance. The cumulative variance contribution was 89.19%, indicating that the model has good discriminative power. These results demonstrated that the PCA model was reliable and could effectively differentiate between the two groups [55]. 

Orthogonal partial least squares discriminant analysis (OPLS-DA) is a supervised method that separates the X matrix into class-related and unrelated components to remove non-discriminatory noise, thereby improving interpretability and classification accuracy. This approach effectively highlights true differences and has been validated for multi-omics integration [56,57]. Consistent with PCA, OPLS-DA also demonstrated clear discrimination of fermented and unfermented samples (Figure 5c). To assess model robustness and avoid overfitting, the OPLS-DA model was validated using cross-validation and response permutation testing. The OPLS-DA model showed a high goodness of fit (R^2^cum = 0.998), while model robustness was supported by cross-validation and permutation testing, yielding a negative Q^2^ intercept (–0.39). 

Volcano plot analysis (Figure 5d) was further conducted to compare metabolite profiles between fermented and unfermented samples. Differential features were screened using the criteria of *p* < 0.05 and fold change > 3. In the fermented sample, a total of 1482 features showed significant alterations, with 912 upregulated and 570 downregulated. These widespread metabolite changes indicated substantial metabolic reprogramming associated with the fermentation process and may be a potential reason for the enhanced antioxidant capacity after fermentation. 

Upregulated features were further scrutinized using a Level 1 annotation confidence scoring system (retention time drift ±18 s; fragmentation score ≥ 45). All features were identified based on relative quantification using normalized LC–MS peak intensities. Features with a *p*-value < 0.05 and a fold change (FC) ≥ 8.0 or ≤1/8.0 were considered significantly altered and are summarized in Table 1. 

Mapping to PubChem databases was applied solely as a technical requirement for target prediction, rather than as a biological selection criterion. Initial screening yielded 25 candidate compounds successfully mapped to PubChem databases. Three compounds (ximoprofen, mebeverine acid, and PGF3alpha-1,15-lactone) were excluded due to biological implausibility within a botanical matrix or lack of biosynthetic rationale and were therefore classified as potential exogenous signals or mis-annotations. Consequently, 22 candidate compounds were retained for subsequent network pharmacology analysis.

### 3.6. PPI Analysis

To explore potential molecular targets associated with the hepatoprotective effects of 1002S, a network pharmacology strategy was employed as an exploratory, systems-level analysis. Targets related to oxidative stress, liver injury, acute immune liver injury, and ferroptosis were retrieved from the GeneCards database using relevance score-based filtering. These categories were selected to reflect pathophysiologically relevant processes in acetaminophen-induced liver injury (AILI).

**Figure 6:**
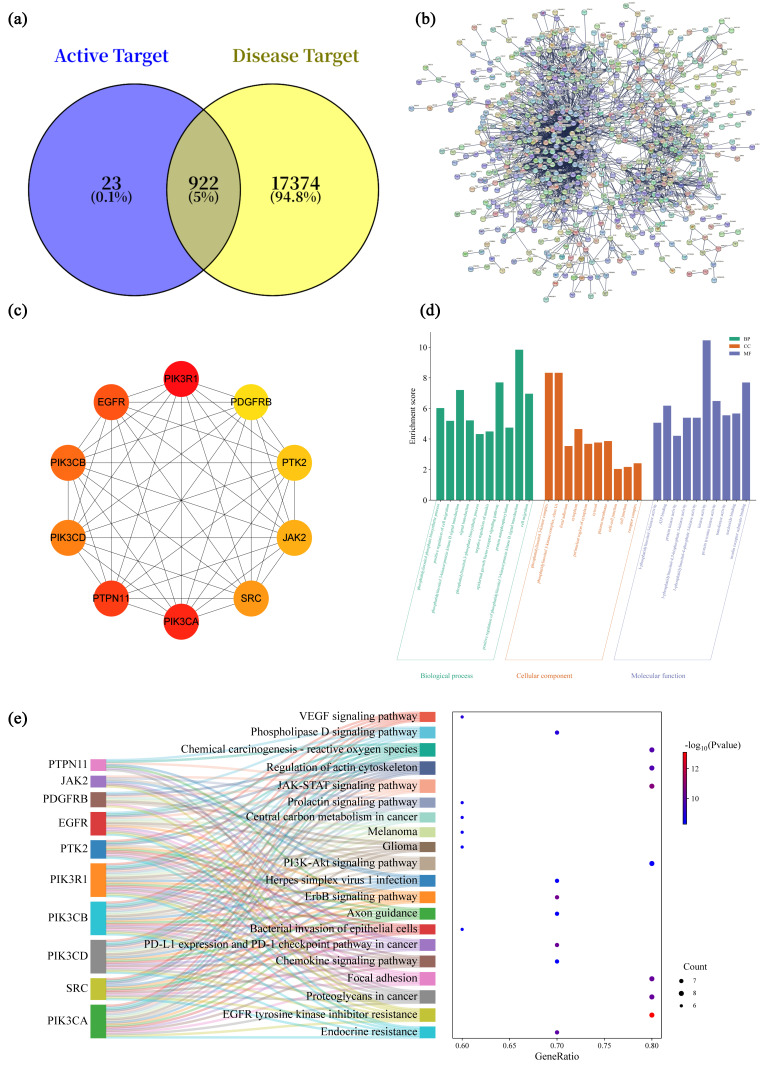
PPI network analysis of potential antioxidant targets of 1002S. (**a**) Venn diagram showing the intersection of antioxidant-related targets; (**b**) PPI network of potential antioxidant targets; (**c**) top 10 predicted core targets ranked by MCC score; (**d**) GO enrichment analysis; (**e**) KEGG pathway enrichment analysis.

Potential targets for 22 selected fermentation-associated compounds were predicted using the SwissTargetPrediction database. After removing duplicate values, 945 potential compound-related targets were obtained. Intersection analysis between disease-associated targets and compound-related targets yielded 922 overlapping genes (Figure 6a), which were considered candidate targets potentially involved in the observed hepatoprotective effects.

These overlapping targets were imported into the STRING database to construct a PPI network. The network consisted of 919 nodes and 2691 edges, with an average node degree of 5.86, indicating extensive interconnectivity among the candidate targets (Figure 6b). The network data were further imported into Cytoscape 3.8.0, and the CytoHubba plugin was applied using the MCC (Maximal Clique Centrality) algorithm to identify the top 10 hub genes [58]. The hub genes identified were PIK3R1, PDGFRB, PTK2, JAK2, SRC, PIK3CA, PTPN11, PIK3CD, PIK3CB, and EGFR (Figure 6c). These hub targets represent central nodes within the interaction network and were selected for subsequent functional enrichment analysis.

### 3.7. GO and KEGG Enrichment Analysis

Functional enrichment analysis of the 10 targets was conducted using the DAVID database [59]. A total of 85 GO functional enrichments were identified, including 25 biological processes (BPs), 17 cellular components (CCs), and 43 molecular functions (MFs). The top 10 targets in each category were visualized (Figure 6d), where "gene count" represents the number of targets enriched in each pathway. 

In the BP category, enriched terms were primarily related to cellular responses to stress, signal transduction, and regulation of cell survival, including phosphatidylinositol phosphate biosynthetic process, phosphatidylinositol 3-kinase (PI3K)/protein kinase B signal transduction, and positive regulation of cell migration. In the CC category, the enrichment results indicate that PI3K complex, focal adhesion and cytoplasmic membrane suggest potential involvement of membrane-associated signaling platforms. Regarding the MF category, kinase-related activities, including phosphatidylinositol kinase activity, protein tyrosine kinase activity, and ATP binding, were prominently represented, reflecting the signaling-centric nature of the identified network.

KEGG pathway enrichment analysis was performed via the DAVID platform, revealing 114 enriched signaling pathways. The top 20 pathways were visualized using a Sankey diagram (Figure 6e) to illustrate the associations between genes and pathways, the significance of *p*-values, and the number of enriched targets. Pathways related to the PI3K-Akt and JAK-STAT signaling pathways were enriched at the predicted target level. These pathways were widely reported to participate in oxidative stress responses, inflammatory regulation, and cell survival in liver injury contexts [60,61].

The GO and KEGG enrichment results represented hypothesis-generating associations rather than definitive mechanistic evidence. The enrichment of the PI3K–Akt and JAK–STAT pathways likely reflected their central roles in stress-responsive signaling networks, rather than indicating pathway-specific activation by individual metabolites. In the context of AILI, excessive reactive oxygen species production triggered widespread oxidative stress and secondary inflammatory responses [5]. The PI3K–Akt signaling pathway was associated with the regulation of the GPX4/SLC7A11 system and Nrf2-related antioxidant responses under oxidative stress conditions [62]. The JAK-STAT pathway exerted a hepatoprotective effect by regulating the expression of anti-inflammatory and antioxidant genes [63]. Therefore, the network pharmacology results provide a systems-level framework that complements experimental observations, rather than serving as causal proof of specific signaling mechanisms.

### 3.8. Molecular Docking and Simulation

Molecular docking simulations were performed as an in silico, hypothesis-generating approach to explore potential interactions between fermentation-derived metabolites and selected proteins associated with antioxidant defense and redox regulation. Docking analysis estimated favorable binding conformations and relative binding energies, which may provide supportive information for candidate compounds, rather than direct evidence of biological activity. Lower binding energies indicated more stable predicted ligand–protein interactions [64,65]. Based on prior experimental data, GPX4, Nrf2, SOD2, and SLC7A11 were selected as Nrf2-associated or redox-related proteins for exploratory docking analysis. AutoDock Vina software was applied to assess the binding energies of 25 metabolites identified from 1002S with the four targets. 

**Figure 7:**
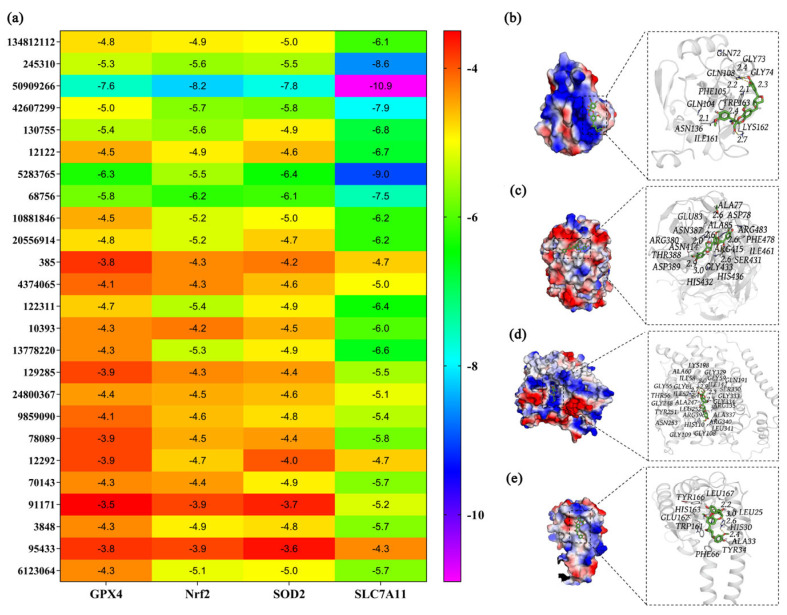
(**a**) Predicted binding energies from molecular docking analysis of 25 metabolites in 1002S with GPX4, Nrf2, SOD2, and SLC7A11. (**b**–**e**) Predicted binding modes of PubChem 50909266 (hemsleyanoside) with GPX4, Nrf2, SOD2, and SLC7A11, respectively. Docking results are presented as supportive, hypothesis-generating evidence and do not indicate direct protein activation.

Figure 7a shows that multiple metabolites exhibited favorable predicted binding energies with four proteins. Approximately 13 metabolites displayed binding energy ≤ −6.0 kcal/mol. Among them, PubChem:50909266 (hemsleyanoside) exhibited the most favorable overall docking scores across the four proteins. These predicted binding energies were −7.6 (GPX4), −8.2 (Nrf2), −7.8 (SOD2), and −10.9 kcal/mol (SLC7A11).

Binding-mode analysis suggested that hemsleyanoside could be accommodated within putative interaction regions of these proteins (Figure 7b–e), forming stabilizing interactions such as hydrogen bonds, hydrophobic contacts, and electrostatic forces. The predicted interactions involved specific amino acid residues on each protein: GLN72, ILE161, and GLN104 on GPX4; ALA77 and ASN389 on Nrf2; GLY109 and GLY329 on SOD2; and LEU167 and ALA33 on SLC7A11. Notably, docking to Nrf2 was conducted as an exploratory analysis, given that Nrf2 was not a classical ligand-binding protein and was primarily regulated through KEAP1-dependent mechanisms. Therefore, these docking results did not imply direct activation of Nrf2, but rather support the potential involvement of Nrf2-associated regulatory networks. 

At present, few studies have characterized the functions of hemsleyanoside [66], and no associated pathways have been experimentally validated. Thus, the docking results should be interpreted as supportive evidence for prioritizing candidate metabolites for further investigation.

Collectively, the target docking analysis suggested that hemsleyanoside may be associated with redox- and ferroptosis-related protein networks, supporting its prioritization for future experimental validation rather than serving as definitive proof of mechanism.

## 4. Discussion

In this study, we demonstrate that fermentation of *D. officinale* flower extract by *S. cerevisiae* is associated with enhanced antioxidant and hepatoprotective effects against AILI. Although *D. officinale* is well recognized for its antioxidant and hepatoprotective properties [33], its therapeutic efficacy may be influenced by the complexity and composition of its bioactive constituents. Our findings indicate that fermentation significantly alters the metabolic profile of *D. officinale* flower extract, which may contribute to the observed enhancement in biological activity, rather than directly demonstrating improved bioavailability.

In vitro antioxidant assays showed that 1002S exhibited relatively higher antioxidant activity than DOFE. In the AILI mouse model, reduced hepatocellular necrosis was observed by H&E staining. The decreased levels of serum ALT, serum AST, and MDA and increased activities of SOD, CAT, GSH, and GSH-Px clearly indicated that 1002S was associated with stronger hepatoprotective effects compared with DOFE. Furthermore, increased expression of Nrf2, SOD2, KEAP1, SLC7A11, and GPX4-associated proteins suggested that 1002S enhanced endogenous antioxidant defense capacity. These findings supported the association between 1002S and modulation of Nrf2-related antioxidant signaling, which may contribute to improved resistance against oxidative stress. However, given the absence of direct ferroptosis-specific endpoints, such as iron accumulation and lipid peroxidation products, the present results did not constitute direct evidence of ferroptosis suppression.

Untargeted metabolomics analysis revealed significant differences in metabolic composition between the fermented (1002S) and unfermented (DOFE) extracts. Integration with network pharmacology analysis suggested that fermentation-associated metabolites are linked to multiple signaling pathways, including the PI3K/Akt and JAK/STAT signaling pathways, which are known to participate in cellular antioxidant defenses, iron metabolism, and cell survival [62,63]. These analyses were conducted as hypothesis-generating approaches, providing a systems-level perspective rather than confirmatory mechanistic evidence.

Molecular docking analysis further selected hemsleyanoside as a candidate metabolite with predicted interactions across multiple redox- and ferroptosis-associated proteins. Given the limited existing knowledge regarding the biological functions of hemsleyanoside [66], these in silico findings should be interpreted as supportive evidence for future investigation, rather than definitive proof of a molecular mechanism. Further experimental studies are required to elucidate its precise biological roles and contribution to hepatoprotection. 

## 5. Conclusions

This study demonstrates that yeast fermentation of *Dendrobium officinale* flower extract (1002S) is associated with enhanced antioxidant and hepatoprotective effects compared with the unfermented extract (DOFE). In AILI models, 1002S exhibited stronger protective effects, as evidenced by improved liver histopathology, reduced serum transaminase levels, and enhanced antioxidant-related responses.

Fermentation markedly altered the metabolic composition of *D. officinale* flower extract, and these changes were accompanied by modulation of redox-related molecular markers, including Nrf2-associated antioxidant proteins. Although ferroptosis-specific endpoints were not directly assessed, the observed molecular changes suggest an association with ferroptosis-related defense mechanisms under oxidative stress conditions.

Collectively, these findings indicate that yeast fermentation represents a promising strategy to enhance the biological activity of *D. officinale*. This work provides experimental evidence supporting the potential development of fermented *D. officinale* flower extract as a functional ingredient or natural adjunct for liver health while highlighting the need for further studies to clarify its pharmacokinetics and mechanistic pathways.

## Figures and Tables

**Table 1:** List of 25 significantly upregulated differential metabolites between the 1002S and DOFE groups.

No	PubChem	Metabolites	Class	VIP	log2 Fold Change	Fold Change	*p*-Value	q-Value
1	134812112	12,13,15-trihydroxy-9E-octadecenoic acid	Fatty acyls	1.3435	3.1378	8.8016	5.00 × 10^−4^	1.10 × 10^−3^
2	245310	8alpha-8-Hydroxy-12-oxo-13-abieten-18-oic acid	Prenol lipids	1.6811	4.8069	27.9916	2.00 × 10^−4^	6.00 × 10^−4^
3	50909266	Hemsleyanoside	Furanoid lignans	1.7153	4.6457	25.0325	1.03 × 10^−5^	2.90 × 10^−5^
4	42607299	PGF3alpha-1,15-lactone	Fatty acyls	1.6644	4.3060	19.7803	3.08 × 10^−6^	9.48 × 10^−6^
5	130755	3-(3-Hydroxy-4-methoxynaphthalen-1-yl)oxypropane-1,2-diol	Naphthalenes	1.4407	3.1657	8.9737	3.82 × 10^−7^	1.42 × 10^−6^
6	12122	3-Hydroxyphenylacetic Acid	Phenols	1.7477	4.6211	24.6081	1.47 × 10^−7^	6.00 × 10^−7^
7	5283765	Calicoferol D	Prenol lipids	2.1178	6.7568	108.1397	4.43 × 10^−8^	2.02 × 10^−7^
8	68756	Ximoprofen	Phenylpropanoic acids	1.7087	4.3921	20.9962	3.11 × 10^−8^	1.47 × 10^−7^
9	10881846	N-3-oxo-tetradec-7(Z)-enoyl-L-Homoserine lactone	Carboxylic acids and derivatives	1.7910	4.8150	28.1486	1.97 × 10^−8^	9.89 × 10^−8^
10	20556914	Mebeverine Acid	Benzene and substituted derivatives	2.3699	8.3774	332.5344	4.47 × 10^−9^	2.59 × 10^−8^
11	385	Pimelic Acid	Fatty acyls	1.5929	3.7665	13.6088	2.49 × 10^−10^	2.04 × 10^−9^
12	4374065	7-oxo-11-Dodecenoic acid	Fatty acyls	1.6233	3.8954	14.8807	1.13 × 10^−11^	1.37 × 10^−10^
13	122311	6-Hydroxy-5-methoxy-1h-indole-2-carboxylic acid	Indoles and derivatives	1.6892	4.2145	18.5642	2.28 × 10^−12^	3.53 × 10^−11^
14	10393	Tyrosol	Phenols	2.2033	7.1688	143.8856	1.99 × 10^−12^	3.16 × 10^−11^
15	13778220	Talaromycin A	Organooxygen compounds	1.5046	3.3383	10.1143	1.33 × 10^−13^	3.18 × 10^−12^
16	129285	Isovalerylalanine	Carboxylic acids and derivatives	1.8612	5.1080	34.4869	9.51 × 10^−14^	2.41 × 10^−12^
17	24800367	Gallicynoic acid G	Hydroxy acids and derivatives	1.5064	3.3454	10.1639	5.33 × 10^−14^	1.51 × 10^−12^
18	9859090	3,10-dihydroxydecanoic acid	Hydroxy acids and derivatives	1.7777	4.6580	25.2463	1.71 × 10^−14^	5.89 × 10^−13^
19	78089	3-Hydroxy-4-Methoxybenzyl Alcohol	Phenols	1.8232	4.8982	29.8191	1.28 × 10^−14^	4.63 × 10^−13^
20	12292	3-Methyladipic acid	Fatty acyls	1.6919	4.2190	18.6222	1.24 × 10^−14^	4.52 × 10^−13^
21	70143	N-Phenethylacetamide	Carboxylic acids and derivatives	2.5221	9.3728	662.9541	4.45 × 10^−15^	2.00 × 10^−13^
22	91171	4-Methyl-1,2-Dihydroxypentane	Organooxygen compounds	1.6525	4.0234	16.2612	4.20 × 10^−15^	1.91 × 10^−13^
23	3848	DL-3-Phenyllactic acid	Phenylpropanoic acids	1.7820	4.6784	25.6053	1.25 × 10^−15^	7.41 × 10^−14^
24	95433	2-Hydroxy-2-Methyl-Butyric Acid	Fatty acyls	1.6945	4.2303	18.7696	1.18 × 10^−15^	7.22 × 10^−14^
25	6123064	Cucurbic acid	Fatty acyls	1.4344	3.0302	8.1691	1.00 × 10^−16^	1.12 × 10^−14^

## Data Availability

The original contributions presented in this study are included in the article/Supplementary Materials. Further inquiries can be directed to the corresponding authors.

## Supplementary Materials

The following supporting information can be downloaded at: https://www.mdpi.com/article/10.3390/cimb48030242/s1.
