# Supplementary material for: Metabolomics and Network Pharmacology-Based Screening of Candidate Hepatoprotective Metabolites in Fermented Dendrobium officinale Against Acetaminophen-Induced Liver Injury
Source: Curr Issues Mol Biol. 2026 Feb 25;48(3):242. doi: 10.3390/cimb48030242 (PMC13026016; doi:10.3390/cimb48030242)

Table S1. Primer sequences list.

NRF2-F	CTTTAGTCAGCGACAGAAGGAC
NRF2-R	AGGCATCTTGTTTGGGAATGTG
Gpx4-F	GCACATGGTCTGCCTGGATAAG
Gpx4-R	TCTTGATTACTTCCTGGCTCCTG
GAPDH-F	CATCACTGCCACCCAGAAGACTG
GAPDH-R	ATGCCAGTGAGCTTCCCGTTCAG

Figure S1. Base peak chromatograms (BPI) of six biological replicates from the DOFE group (panels a–f) and six from the 1002S group (panels g–l) analyzed by LC-MS in positive ion mode.

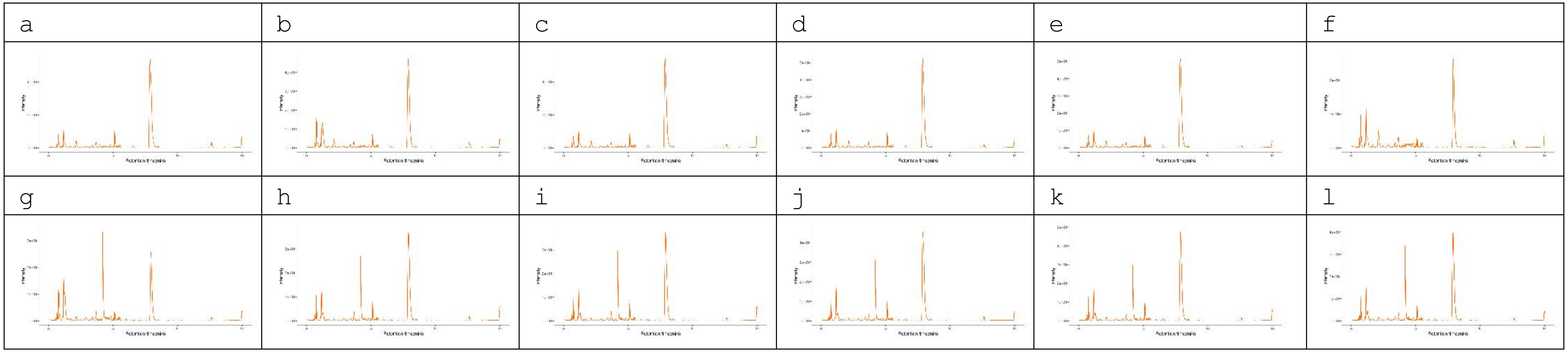

Figure S2. Base peak chromatograms (BPI) of six biological replicates from the DOFE group (panels a–f) and six from the 1002S group (panels g–l) analyzed by LC-MS in negative ion mode.

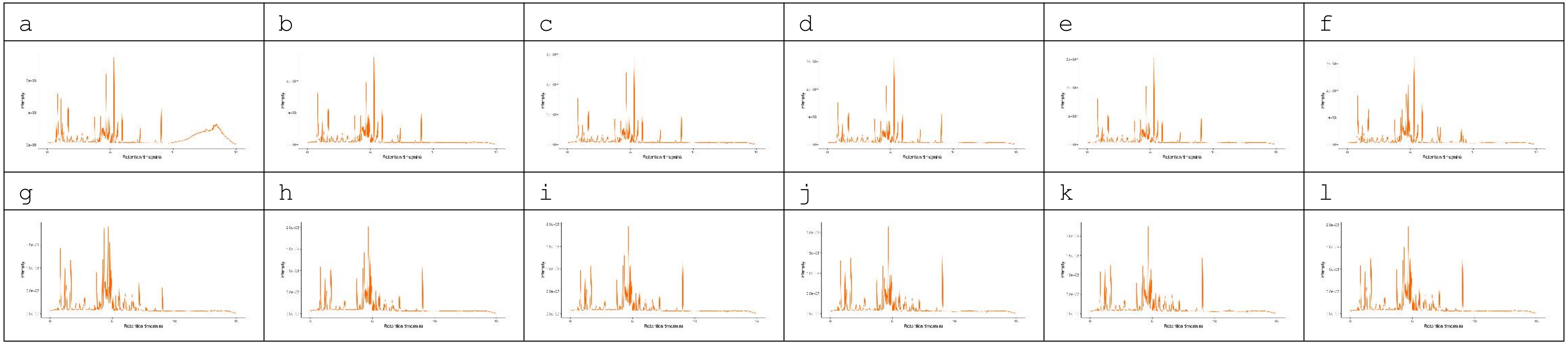

Figure S3. Total ion chromatograms (TIC) of six biological replicates from the DOFE group (panels a–f) and six from the 1002S group (panels g–l) analyzed by GC-MS.

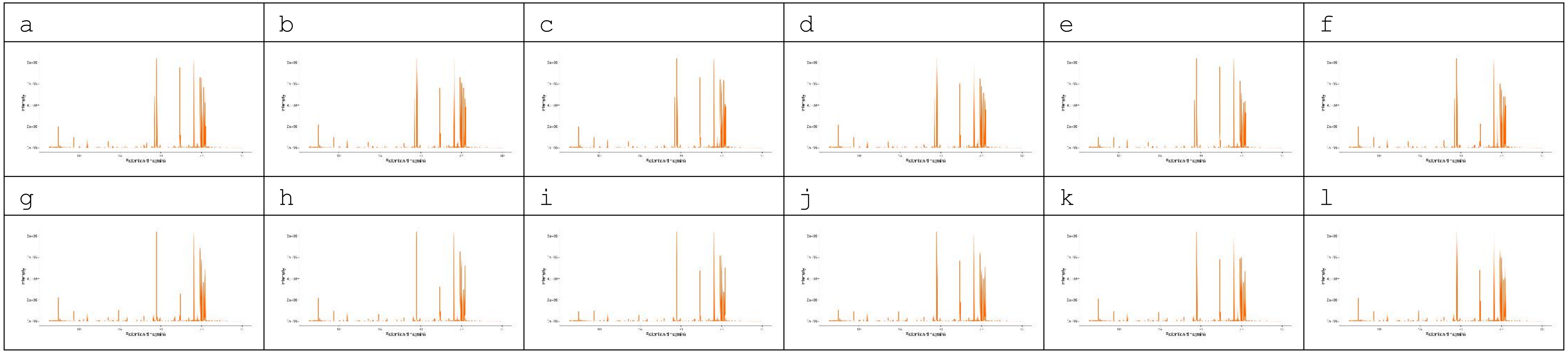

Supplement: Supplementary file 1 [file cimb-48-00242-s001.zip › cimb-4110451-supplementary.pdf]
